# Biological interactions of *CYP2C19* genotypes with CYP3A4*18, CYP3A5*3, and *MDR1-3435* in living donor liver transplantation recipients

**DOI:** 10.1186/2047-1440-2-6

**Published:** 2013-04-23

**Authors:** King-Wah Chiu, Tsung-Hui Hu, Toshiaki Nakano, Kuang-Den Chen, Chia-Yun Lai, Li-Wen Hsu, Hui-Peng Tseng, Ho-Ching Chiu, Yu-Fan Cheng, Shigeru Goto, Chao-Long Chen

**Affiliations:** 1 Liver Transplant Program, Kaohsiung Chang Gung Memorial Hospital, Chang Gung University, College of Medicine, Kaohsiung, Taiwan; 2 Kaohsiung Chang Gung Memorial Hospital, Chang Gung University College of Medicine, 123 Ta-Pei Road, Niao-Sung, Kaohsiung, 833, Taiwan

**Keywords:** Living donor liver transplantation, Cytochrome P450, CYP3A4*18, CYP3A5*3, *MDR1-3435*, *CYP2C19* genotypes

## Abstract

**Background:**

Polymorphisms in *CYP2C19* are related to the metabolic oxidation of drugs to varying degrees. The CYP3A4*18, CYP3A5*3, and *MDR1-3435* variant alleles are very important, particularly in tacrolimus metabolism in organ transplant rejection.

**Aim:**

The aim of this study is o explore possible interactions among different *CYP2C19* genotypes, namely, between homozygous extensive metabolizers (HomEM), heterozygous extensive metabolizers (HetEM), and poor metabolizers (PM), and the CYP3A4*18, CYP3A5*3, and *MDR1-3435* variants in living donors and patients who received a living donor liver transplant (LDLT).

**Methods:**

This prospective study enrolled 133 living donors and 133 corresponding recipients. On the basis of the HomEM, HetEM, and PM *CYP2C19* genotypes, the distributions of CYP3A4*18 (exon 10; T878C), CYP3A5*3 (intron 3; A6986G), and *MDR1-3435* (exon 26; C3435T) genotypes were analyzed for single nucleotide polymorphisms among donors and recipients.

**Results:**

Among 102 HomEM genotypes, including 56 donors and 46 recipients, 91.2% of individuals harbored the T/T genotype of CYP3A4*18; 53.9% possessed G/G, and 34.3% had A/G genotypes of CYP3A5*3; and 38.2% had C/C and 50.0% had C/T genotypes at *MDR1-3435*. Among 130 HetEM genotypes, including 58 donors and 72 recipients, 97.7% of individuals possessed T/T genotype at CYP3A4*18; 50.0% harbored G/G and 41.5% had A/G genotypes at CYP3A5*3; and 40.0% had C/C and 49.2% had C/T genotypes at *MDR1-3435*. In 34 PMs, including 19 donors and 15 recipients, 88.2% had T/T genotypes at CYP3A4*18; 41.2% had G/G and 58.8% had A/G genotypes at CYP3A5*3; and 47.1% possessed C/C and 47.1% had C/T genotypes at *MDR1-3435*. On the basis of the *CYP2C19* genotypes, no statistically significant distribution of genotypes were observed between donors and recipients for all genotypes of CYP3A4*18, CYP3A5*3, and *MDR1-3435* (*P* >0.05).

**Conclusions:**

In conclusion, the *CYP2C19* genotypes do not affect the expression of CYP3A4*18, CYP3A5*3, or *MDR1-3435* variants, which are independently distributed among donors and recipients during LDLT.

## Introduction

Cytochrome P450 in the liver is one of the key enzyme complexes in the primary drug-metabolizing system in humans. Recipients of living-donor liver transplantation (LDLT) exhibit interesting biological distributions of *CYP2C19* genotypes that differ between the recipient’s tissue and the newly grafted tissue [1]. Acute rejection or abnormal postoperative liver function after LDLT could result from complications in cytochrome P450 function [2,3]. We have recently reported a homogenous phenomenon observed in *CYP2C19* genotypes [4]. Anti-rejection agents such as tacrolimus usually target *CYP3A4*, *CYP3A5*, and *MDR-1*, which are the major metabolic isoenzymes of cytochrome P450. In the present study, we aimed to investigate the *CYP2C19* genotypes, which have been classified as homozygous extensive metabolizers (HomEM), heterozygous extensive metabolizers (HetEM), and poor metabolizers (PM), and to identify any interactions between the CYP3A4*18, CYP3A5*3, and *MDR1-3435* variants by characterizing differences between the genotype distribution of healthy living donors and patients with liver disease who received LDLT.

## Methods

This present study included 133 living donors and 133 consecutive LDLT recipients who were enrolled in our transplantation program. The mean age of the recipients was 42.62 years (range, 0.6 to 68 years), including 98 male and 35 female subjects. The etiologies for LDLT are shown in Table 1. There were 60 (45.1%) recipients with hepatitis B-related end-stage liver disease (ESLD), of which 35 (58.3%) were associated with hepatocellular carcinoma (HCC); 22 (16.5%) recipients with hepatitis C-related ESLD, including 11 (50.0%) with HCC; 9 (6.8%) recipients with both hepatitis B- and C-related ESLD, including 5 (55.6%) with HCC; 8 (6.0%) recipients with alcoholic-related ESLD, including 2 (25%) with HCC; and 23 pediatric recipients with biliary atresia. The remaining 11 recipients had autoimmune liver disease (1 patient), Alagille syndrome (3 patients), portal vein occlusion (1 patient), primary biliary cirrhosis (2 patients), polycystic liver (1 patient), or cryptogenic liver cirrhosis (3 patient). Complete liver function was assessed on day 1 after liver transplantation (D1) and on day 30 after liver transplantation (D30), including measurements of alanine transferase (ALT), aspartate transferase (AST), total bilirubin (T-Bil), prothrombin time (PT) with international normal range (INR), and albumin (Alb). Donors and recipients were classified on the basis of the *CYP2C19* genotype, as detailed in our previous report [1]. HomEMs were individuals with wild-type alleles (genotype: *1/*1), whereas HetEMs were those who possessed a single mutated allele (genotype: *1/*2 or *1/*3). Patients with homozygous mutations (m1 in exon 5 or m2 in exon 4) of *CYP2C19* (genotype: *2/*2, *3/*3, or *2/*3) were defined as PMs. This study comprised a total of 102 HomEMs, including 56 donors and 46 recipients; 130 HetEMs, including 58 donors and 72 recipients; and 34 PMs, including 19 donors and 15 recipients. On the basis of the *CYP2C19* genotype classification, HomEM, HetEM, or PM individuals were analyzed to test for carrier status of other known single nucleotide polymorphism (SNP) variant alleles to determine biological distributions of CYP3A4*18 (exon 10; T878C), CYP3A5*3 (intron 3; A6986G), and *MDR1-3435* (exon 26; C3435T). Genomic DNA was extracted from peripheral leukocytes and genotyped for evaluating the *CYP2C19*, CYP3A4*18, CYP3A5*3, and *MDR1-3435* status by polymerase chain reaction-restriction fragment length polymorphism (PCR-RFLP) and direct sequencing analysis.

**Table 1:** Etiology of the 133 study subjects who required living donor liver transplantation

**Category**	**Recipient** **N (%)**	**Associated with HCC** **N (%)**
HBV	60 (45.1)	35 (58.3)
HCV	22 (16.5)	11 (50.0)
HBV + HCV	9 (6.8)	5 (55.6)
Alcoholic	8 (6.0)	2 (25.0)
Biliary atresia	23 (17.3)	
Other^a^	11 (8.3)	
Total	133 (100)	53 (39.9)

^a^autoimmune (1), Alagille syndrome (3), portal vein occlusion (1), primary biliary cirrhosis (2), polycystic liver (1), and cryptogenic liver cirrhosis (3).

HBV, hepatitis B virus; HCC, hepatocellular carcinoma; HCV, hepatitis C virus.

### Genotyping of *CYP3A4*, *CYP3A5*, and *MDR1-3435*

Polymerase chain reaction/ligase detection reaction assay (PCR/LDR) was employed for genotyping the CYP3A4*18B and CYP3A5*3 SNPs. The PCR conditions consisted of a denaturation step at 95°C for 15 min, followed by 35 cycles of 94°C for 30 s, 65°C for 1 min, and 72°C for 1 min, followed by a final extension step at 72°C for 7 min. The specific amplified fragments were used in an LDR assay to identify the mutations associated with CYP3A4*18B and CYP3A5*3. The LDR assay was performed as follows: 10 μL of the reaction mix contained 1 μL of 1× ligase reaction buffer (New England Biolabs, USA), 1 μL of probes (12.5 pmol/μL each), 0.05 μL (2 U) of thermostable Taq DNA ligase (New England Biolabs), and 1 μL of PCR product. The ligation reaction was performed with a GeneAmp PCR System 9600 (Perkin Elmer, USA) as follows: 15 min at 95°C, followed by 35 cycles of 30 s at 94°C and 2 min at 60°C. The products were separated by agarose gel electrophoresis and analyzed with an ABI PRISM 377 DNA sequencer [5]. Genotyping was performed using an independent external contractor (Biowing Applied Biotechnology Co. Ltd., China). Genomic DNA was isolated from whole blood using the UltraPure*™* Genomic DNA Isolation Kit (Shanghai SBS Genetech Technology Co., China). PCR-RFLP was performed to genotype exon 26 (C3435T) variant alleles in the *MDR1* gene, with slight modifications. The PCR conditions consisted of a denaturation step at 95°C for 5 min, followed by 30 cycles of denaturation at 94°C for 30 s, annealing at 54°C to 59°C for 50 s, and elongation at 72°C for 1 min, followed by a final extension at 72°C for 10 min. PCR products were digested with *Dpn*II (C3435T) and analyzed by electrophoretic separation on agarose gels, followed by direct visualization over an ultraviolet transilluminator after ethidium bromide staining [6].

This work was supported by a grant from Chang Gung Memorial Hospital (CMRPG8A0631 to K-WC) of Taiwan, which also granted ethical approval to our study. This study was approved by the Institutional Review Board (IRB), and informed consent was obtained from participants or from a parent or guardian in case of minor participants.

### Statistical analyses

Statistical analyses were performed using SPSS software (version 12.0; SPSS, Chicago, IL, USA). Parameters between HomEM or HetEM and PMs in donors and recipients were compared using the *X*^2^ test, Fisher’s exact test, and Student’s *t*-test. *P* values less than 0.05 were considered statistically significant.

## Results

In this study, 68.4% (91/133) of ESLD cases were virus-related, including 45.1% (60/133) of hepatitis B cases, 16.5% (22/133) of hepatitis C cases, and 6.8% (9/133) of cases with both hepatitis B and C. Furthermore, 39.9% (53/133) recipients had HCC. Excluding the 23 biliary atresia and 3 Alagille syndrome pediatric cases, 49.5% (53/107) of adult recipients had HCC (Table 1). Clinical profiles of the 133 donors and 133 recipients on the day of the LDLT operation are shown in Table 2. The mean age of the donor population was significantly younger than the recipient population (30.27 years (range 18 to 53 years) versus 42.62 years (range 0.6 to 68 years); *P* <0.001). On D1 of liver transplantation, all donors showed significantly better results of all liver function tests than did the recipients, including ALT (14.43 ± 14.75 versus 252.35 ± 446.40; *P* <0.001), AST (18.3 ± 7.19 versus 292.42 ± 660.86; *P* <0.001), T-Bil (0.64 ± 0.57 versus 7.19 ± 11.71; *P* <0.001), PT (INR) (0.97 ± 0.09 versus 1.91 ± 3.34; *P* = 0.003), and Alb (4.24 ± 0.79 versus 3.06 ± 0.88; *P* <0.001). On D30, after LDLT, all liver function tests of the recipients were significantly better than those on D1, including ALT (252.43 ± 446.40 versus 82.40 ± 244.43, *P* <0.001), AST (292.42 ± 660.86 versus 65.54 ± 300.20, *P* <0.001), T-Bil (7.19 ± 11.71 versus 1.24 ± 3.60, *P* <0.001), PT (INR) (1.91 ± 3.34 versus 1.05 ± 0.40, *P* <0.001), and Alb (3.06 ± 0.88 versus 3.74 ± 0.95, *P* <0.001).

**Table 2:** Clinical profiles of 133 donors and 133 recipients for living donor liver transplantation and comparison of their clinical profiles on day 1 (D1) and day 30 (D30) after transplantation

**Category**	**Donor N = 133**	**Recipient N = 133**		***P *value**		
Age (mean) (range)	30.27 (18 to 53)	42.62 (0.6-68)		<0.001		
Sex M:F	83:50	98:35		0.292		
		*D1*	*D30*	D:R*D1*	D:R*D30*	R*D*1:R*D30*
ALT	14.43 ± 14.75	252.35 ± 446.40	82.40 ± 244.43	<0.001	0.003	<0.001
AST	18.3 ± 7.19	292.42 ± 660.86	65.54 ± 300.20	<0.001	0.087	<0.001
T-Bil	0.64 ± 0.57	7.19 ± 11.71	1.24 ± 3.60	<0.001	0.071	<0.001
PT (INR)	0.97 ± 0.09	1.91 ± 3.34	1.05 ± 0.40	0.003	0.025	<0.001
Alb	4.24 ± 0.79	3.06 ± 0.88	3.74 ± 0.95	<0.001	0.148	<0.001
Tacrolimus (ng/mL) (n = 107)		2.51 ± 2.73	6.17 ± 9.58			<0.001
cyA (ng/mL) (n = 26)		283.89 ± 308.93	1058.30 ± 582.37			<0.001

D, donor; D1, post liver transplantation day 1; D30, post liver transplantation day 30; R, recipient.

On the day of LDLT, 107 adult recipients received tacrolimus and 26 pediatric recipients received cyclosporine A (CsA). The serum concentrations of tacrolimus and CsA were 2.51 ± 2.73 ng/mL and 283.89 ± 308.93 ng/mL, respectively, on D1 and 6.17 ± 9.58 ng/mL and 1058.30 ± 582.37 ng/mL, respectively, on D30; these results show that the serum concentrations of both tacrolimus and cyA were significantly higher on D30 than on D1 after LDLT (*P* <0.001; Table 2).

Among the 266 subjects with *CYP2C19* genotypes (comprising 133 healthy donors and 133 diseased recipients), 102 had HomEM genotypes, including 56 donors and 46 recipients. The 2 wild-type CYP3A4*18 genotypes, T/T and T/C genotypes, were distributed as follows: 53.8% (50/93) of individuals with the T/T genotype were donors and 46.2% (43/93) were recipients, and 66.7% (6/9) of cases with the T/C genotype were donors and 33.3% (3/9) were recipients. The 3 CYP3A5*3 genotypes namely, G/G, A/G, and A/A were distributed as follows: 58% (32/55) of individuals with the G/G genotype were donors and 42.0% (23/55) were recipients, 48.6% (17/35) of individuals with the A/G genotype were donors and 51.4% (18/35) were recipients, and 58.3% (7/12) of individuals with the A/A genotype were donors and 41.7% (5/12) were recipients. The 3 *MDR1-3435* genotypes, C/C, C/T, and T/T, were distributed as follows: 59.0% (23/39) of individuals with the C/C genotype were donors and 41.0% (16/39) were recipients, 56.9% (29/51) of individuals with the C/T genotype were donors and 43.1% (22/51) were recipients, and 33.3% (4/12) of individuals with the T/T genotype were donors and 66.7% (8/12) were recipients. There was no statistically significant correlation between healthy donors and diseased recipients bearing the *CYP2C19* HomEM genotype with CYP3A4*18, CYP3A5*3, or *MDR1-3435* polymorphisms (*P* >0.05).

In this study, 130 individuals had the HetEM genotype, including 58 donors and 72 recipients. The 2 wild-type and CYP3A4*18 genotypes were distributed as follows: 45.7% (58/127) of individuals with the T/T genotype were donors and 54.3% (69/93) were recipients, and 0% (0/3) of those with the T/C genotype were donors and 100.0% (3/3) were recipients. The 3 CYP3A5*3 genotypes were distributed as follows: 46.2% (30/65) of individuals with the G/G genotype were donors and 53.8% (35/65) were recipients, 48.0% (26/54) of individuals with the A/G genotype were donors and 52.0% (28/54) were recipients, and 18.2% (2/11) of individuals with the A/A genotype were donors and 81.8% (9/11) were recipients. The 3 *MDR1-3435* genotypes were distributed as follows: 42.3% (22/52) of individuals with the C/C genotype were donors and 57.7% (30/52) were recipients, 50.0% (32/64) of individuals with the C/T genotype were donors and 50.0% (32/64) were recipients, and 28.6% (4/14) of individuals with the T/T genotype were donors and 71.4% (10/14) were recipients. No statistically significant association was observed between healthy donors and diseased recipients with the *CYP2C19* HetEM genotype and the CYP3A4*18, CYP3A5*3, or *MDR1-3435* genotypes (*P* >0.05). The data presented in Table 3 summarizes the negative correlations between the HomEM, HetEM, and PM *CYP2C19* genotypes and the CYP3A4*18, CYP3A5*3, or *MDR1-3435* genotypes. Our data indicate that these genotypes had similar distributions in both donors and recipients, suggesting that there were no statistically significant differences between CYP3A4*18 (T/T and T/C), CYP3A5*3 (G/G, A/G and A/A), and *MDR1-3435* (C/C, C/T and T/T) haplotypes, as well as the different *CYP2C19* HomEM, HetEM, and PM genotypes between healthy donors and recipients with ESLD. Hence, there were independent isoenzymes and no correlation of genetic interaction between *CYP2C19* and CYP3A4*18, CYP3A5*3, or *MDR1-3435* not only, but also the variant haplotypes or genotypes.

**Table 3:** Association between the*CYP2C19*genotypes:CYP3A4*18,CYP3A5*3,and*MDR1-3435*in 133 donors and 133 recipients after living donor liver transplantation

***CYP2C19***	CYP3A4*18^**a**^		CYP3A5*3^**b**^			***MDR1-3435***^**c**^		
**Genotype (n)**	**T/T**	**T/C**	**G/G**	**A/G**	**A/A**	**C/C**	**C/T**	**T/T**
HomEM: n = 102 (%)	93 (91.2)	9 (8.8)	55 (53.9)	35 (34.3)	12 (11.8)	39 (38.2)	51 (50.0)	12 (11.8)
**D: n = 56 (%)**	50 (53.8)	6 (66.7)	32 (58)	17 (48.6)	7 (58.3)	23 (59)	29 (56.9)	4 (33.3)
**R: n = 46 (%)**	43 (46.2)	3 (33.3)	23 (42)	18 (51.4)	5 (41.7)	16 (41)	22 (43.1)	8 (66.7)
HetEM: n = 130 (%)	127 (97.7)^d^	3 (2.3)^d#^	65 (50.0)	54 (41.5)	11 (8.5)	52 (40)	64 (49.2)	14 (10.8)
**D: n = 58 (%)**	58 (45.7)	0 (0)	30 (46.2)	26 (48)	2 (18.2)	22 (42.3)	32 (50)	4 (28.6)
**R: n = 72 (%)**	69 (54.3)	3 (100)	35 (53.8)	28 (52)	9 (81.8)	30 (57.7)	32 (50)	10 (71.4)
PM: n = 34 (%)	30 (88.2)^d^	4 (11.8)^d^	14 (41.2)	20 (58.8)		16 (47.1)	16 (47.1)	2 (5.9)
**D: n = 19 (%)**	16 (53.3)	3 (75)	9 (64.3)	10 (50)	0	11 (68.8)	6 (37.5)	2 (100)
**R: n = 15 (%)**	14 (46.7)	1 (25)	5 (35.7)	10 (50)		5 (31.2)	10 (62.5)	0 (0)

D, donor; HetEM, heterozygous extensive metabolizers; HomEM, homozygous extensive metabolizers; PM, poor metabolizers; R: recipient.; ^a^CYP3A4*18 (exon 10; T878C).

^b^CYP3A5*3 (intron 3; A6986G).

^c^*MDR1-3435* (exon 26; C3435T).

^d^*P* < 0.05. There was no statistically significant difference between the haplotypes of CYP3A4*18 (T/T and T/C), CYP3A5*3 (G/G, A/G, and A/A), and *MDR1-3435* (C/C, C/T, and T/T) and also between the different *CYP2C19* genotypes (HomEM, HetEM, and PM) between healthy donors and recipients with end stage liver disease. There were independent isoenzymes and no correlation of genetic interaction between *CYP2C19* and CYP3A4*18, CYP3A5*3, or *MDR1-3435* not only, but also the variant haplotypes or genotypes.

There were 34 PMs in this cohort, including 19 donors and 15 recipients. The 2 wild-type CYP3A4*18 genotypes were distributed as follows: 3.3% (16/30) of individuals with the T/T genotype were donors and 46.7% (14/30) were recipients, 75.0% (3/4) of individuals with the T/C genotype were donors and 25.0% (1/4) were recipients. Only 2 of the possible CYP3A5*3 genotypes were identified among individuals with PMs: 64.3% (9/14) of individuals with the G/G genotype were donors and 35.7% (5/14) were recipients, and 50.0% (10/20) of individuals with the A/G genotype were donors and 50.0% (10/20) were recipients. No CYP3A5*3 A/A genotypes were detected. The 3 *MDR1-3435* genotypes were distributed as follows: 68.8% (11/16) of individuals with the C/C genotype were donors and 31.2% (5/16) were recipients, 37.5% (6/16) of individuals with the C/T genotype were donors and 62.5% (10/16) were recipients, and 100.0% (2/2) of individuals with the T/T genotype were donors. No statistically significant association was found between healthy donors and diseased recipients either with the *CYP2C19* PM genotype or with the CYP3A4*18, CYP3A5*3, and *MDR1-3435* genotypes (*P* >0.05).

## Discussion

From our previous studies, we know that *CYP2C19* expresses three genotypes with different drug metabolization capacities [1,3]. In this study, we attempted to investigate possible genetic interactions between *CYP2C19* and CYP3A4*18, CYP3A5*3, or MDR1-3435 in detail. In the present study, we focused on the expression of genetic polymorphisms in the CYP3A4*18, CYP3A5*3, and *MDR1-3435* genotypes. No significant differences were found in the distributions between CYP3A4*18 (exon 10; T878C), CYP3A5*3 (intron 3; A6986G), or *MDR1-3435* (exon 26; C3435T) genotypes on the basis of different *CYP2C19* genotypes between healthy liver donors and patients with poor liver function who received LDLT. Although all these proteins are important isoenzymes of cytochrome P450 in drug metabolism in the liver, only the differences in *CYP2C19* genotypes have been documented [1]. CYP3A4*18, CYP3A5*3, and *MDR1-3435* polymorphisms do not appear to have a functional effect following LDLT in either the donor with normal liver function or the recipient with ESLD. A recent study showed that the *CYP2C19* genotype, unlike *MDR1* and *IL-1B* genotypes, had an impact on the efficacy of *Helicobacter pylori* eradication in peptic ulcer patients treated with pantoprazole in triple therapy administrations [7]. In the present study, individuals receiving LDLT who had different *CYP2C19* genotypes (HomEM [41 and 43.1%], HetEM [57.7 and 50%], and PM [31.2 and 62.5%]) did not show different distributions of CYP3A4*18, CYP3A5*3, and *MDR1-3435* genotypes (C/C and C/T). This observation was consistent in both donors and recipients.

**Figure 1:**
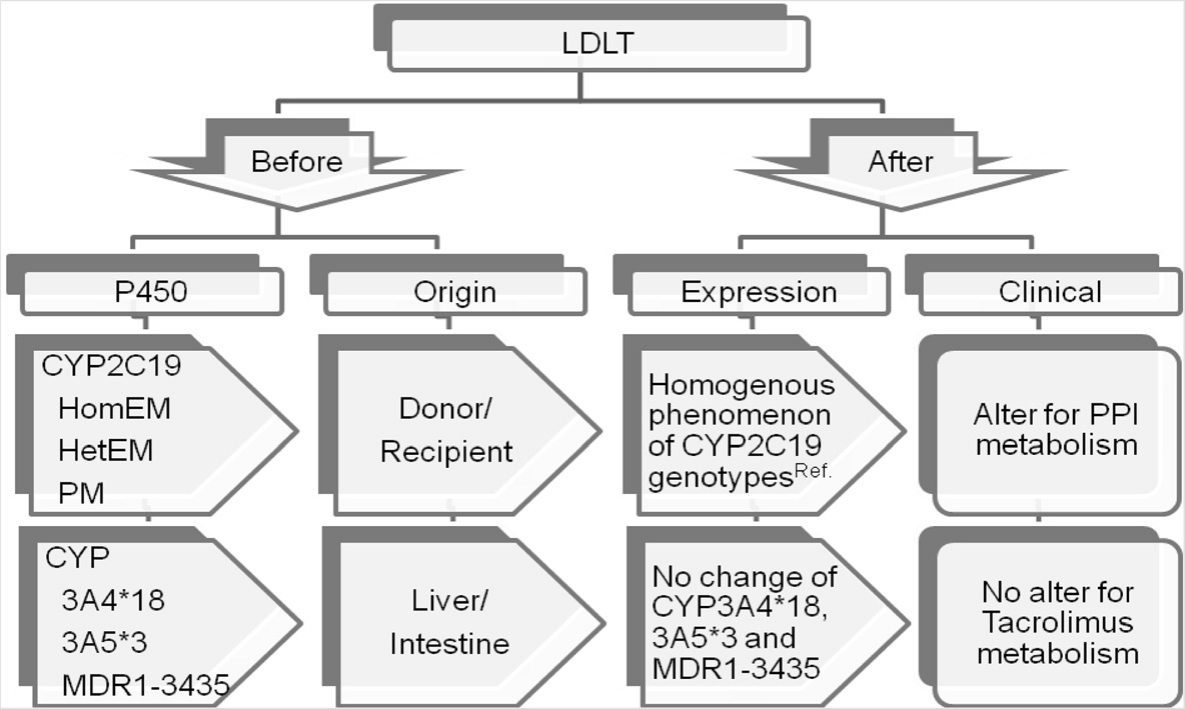
Flow chart of the possible relationship between *CYP2C19 *genotypes and CYP3A4*18, CYP3A5*3, and *MDR1-3435 *genotypes.[4].

After LDLT, the serum levels of immunosuppressive agents were significantly lower on D1 than on D30, whereas the results of all liver functional tests (ALT, AST, T-Bil, PT(INR), and Alb) were significantly higher on D1 than on D30. These results led us to hypothesize that the immunosuppressive agents affected the stability of metabolic enzymes in the cytochrome P450 system. Previous published reports describe *CYP3A* as the most abundant enzyme of the P450 subfamily in the human liver and intestine, accounting for 30% of the total P450 in the human liver, and metabolizing approximately 50% of currently used clinical drugs [8-10]. The impacts of different CYP2C19, CYP3A4*18, CYP3A5*3, and *MDR1-3435* genotypes on LDLT have been outlined in a flow chart presented in Figure 1. In our previous study, the homogenous phenomenon was attributed to the different *CYP2C19* genotypes (HomEM, HetEM, and PM) between an LDLT donor and recipient owing to the uniqueness of the human liver [4]. The metabolism of the proton pump inhibitor (PPI) was dependent on the *CYP2C19* genotypes in the cytochrome P450 system, primarily in the liver. HomEM genotypes were found to better metabolize some drugs than did the HetEM and PM genotypes during LDLT [1]. If the donor possessed a *CYP2C19* PM genotype, the recipient assumed a PM genotype (rather than the original *CYP2C19* HomEM genotype) because of the homogenous phenomenon [4]. In contrast, the CYP3A4*18 and CYP3A5*3 genetic polymorphisms have two origins: the liver and intestine [8,9]. In the postnatal human liver, *CYP3A4* and *CYP3A5* are the two major *CYP3A* enzymes, which have overlapping substrate specificities [10]. When the liver function worsens, the drug metabolic function of the intestine may compensate, leading to unaltered drug metabolism, such as for tacrolimus (Figure 1). Depending on the liver function, the *CYP2C19* genotypes HomEM, HetEM, and PM were more likely to present abnormal postoperative liver function and graft pathology [3]; however, this was not observed in the present study for the CYP3A4*18, CYP3A5*3, and *MDR1-3435* polymorphisms in LDLT. As reported previously, *CYP2C19* genotype expression is not demonstrated well by western blotting [11]. *CYP3A5* protein expression is highly variable in the human liver, in particular, because of the high frequency of a SNP CYP3A5*3 A6986G in an intron [12]. The CYP3A4*18B SNP in intron 10 was first discovered by direct sequencing in a Japanese population. It was speculated that this variant was associated with increased *CYP3A4* activity [5], and this speculation was extended by exploring cyclosporin A (CsA) metabolism in healthy Chinese subjects [6]. A number of SNPs have been identified in the *MDR1* gene by large-scale sequencing. For example, our study probed the C3435T variant in exon 26 [13,14]. The PCR-RFLP method to detect the *MDR1* 3435C/T polymorphism has also been widely used, as was used recently in our study [7]. Although there is no evidence suggesting that intestinal expression of *CYP3A4*, *CYP3A5*, and *MDR-1* play an important role, human cytochrome P450 enzymes have been expressed in *Escherichia coli*. Simplified bacterial systems can explain the possibility of intestinal activation of these enzymes [15]. A previous study has shown that the intestinal mucosa contains prominent forms of cytochrome P450, which are similar to liver cytochrome P450p in their structure, function, and some regulatory characteristics [16].

Therefore, the results of the present study do not impel us to change strategies and to combine certain donors with certain recipients. According to our previous study, evidence for graft rejection occurred within a month after LDLT [2]. In other words, the cytochrome P450 system stabilizes with time, up to 1 month after LDLT. The increase in serum level of immunosuppressive agents is followed by clinical liver functions leading to stability. Whether these studies can be performed in cadaveric liver transplantation cases is presently unclear.

In our cohort, 68.4% of individuals receiving LDLT had underlying chronic viral hepatitis-related ESLD, including 39.9% of individuals who had HCC. There was no difference in CYP3A4*18, CYP3A5*3, and *MDR1-3435* genotypes between the donors and recipients. In addition to the etiology of the underlying disease, age also did not influence the distribution of CYP3A4*18, CYP3A5*3, and *MDR1-3435* genotypes. No remarkable difference was observed between donors and recipients, as well as between pediatric and adult recipients.

From our data, the haplotypes of the CYP3A4*18, CYP3A5*3 or *MDR1-3435* do not seem to correlate with tacrolimus metabolism in these recipients, but the variant stability of these enzymes are significantly different on D1 and D30 after LDLT. Drug levels were lower on D1 and higher on D30, but there were no correlations to the haplotypes of CYP3A4*18, CYP3A5*3, or *MDR1-3435* and/or different genotypes of *CYP2C19* HomEM, HetEM, and PM, except for their stability.

In conclusion, the *CYP2C19* genotypes, HomEM, HetEM, and PM, do not affect the expression of CYP3A4*18, CYP3A5*3, and *MDR1-3435* polymorphisms. These polymorphisms were independently distributed among donors and recipients, as well as healthy and diseased livers, because the source may be located outside the liver during LDLT.

## Abbreviations

Alb: albumin; ALT: alanine transferase; AST: aspartate transferase; DNA: deoxyribonucleic acid; ESLD: end-stage liver disease; HBV: hepatitis B virus; HCC: hepatocellular carcinoma; HCV: hepatitis C virus; HetEM: heterozygous extensive metabolizer; HomEM: homozygous extensive metabolizer; INR: international normal range; IRB: Institutional Review Board; LDLT: living donor liver transplantation; PCR/LDR: polymerase chain reaction/ligase detection reaction assay; PCR-RFLP: polymerase chain reaction-restriction fragment length polymorphism; PM: poor metabolizer; PPI: proton pump inhibitor; PT: prothrombin time; SNP: single nucleotide polymorphism; T-Bil: bilirubin total.

## Competing interests

The authors declare that they have no competing interests.

## Authors’ contributions

KWC and THH contributed in the preparation of this manuscript in terms of literature review and writing the manuscript. CYL, LWH, HPT, and HCC contributed in data collection, data entry, data analysis, interpretation, and drafting of the article. TN, KDC, YFC, SG, and CLC contributed by writing specific sections of the manuscript and in revising it critically for important intellectual content. All authors have read the manuscript and have approved the final version of this manuscript.
